# Acute Effects on Physical Performance Measures after 45 Min of Official Competition in Youth Soccer Players

**DOI:** 10.3390/jfmk6020049

**Published:** 2021-06-04

**Authors:** Federico Gazzo, Julián Giráldez, Rodrigo Villaseca-Vicuña, José Antonio González-Jurado, Santiago Zabaloy

**Affiliations:** 1 Physical Performance and Sports Research Centre, University of Pablo de Olavide, 41013 Sevilla, Spain; fngaz@alu.upo.es (F.G.); jcgir@alu.upo.es (J.G.); rvilvic@alu.upo.es (R.V.-V.); jagonjur@upo.es (J.A.G.-J.); 2 Faculty of Physical Activity and Sports, University of Flores, Buenos Aires 1406, Argentina; 3 Football Federation of Chile, Santiago 7930013, Chile

**Keywords:** testing, physical performance, soccer, fatigue

## Abstract

(1) Background: An improved understanding of soccer players’ match-related physical performance and recovery may help conditioning programs and re-warm up strategies to increase team performance during official competitions. Therefore, the aim of this study was to analyze the acute effects of 45 min of official competition (first half in matches) on physical performance variables in U-16 youth soccer players. (2) Methods: 20 male soccer players (age: 14.4 ± 0.5 years; height: 1.70 ± 0.05 cm; body mass: 65.1 ± 11.6 kg) were recruited to participate in this study. Data was collected from five official matches. Participants performed the assessments in two stages of each match: after the pre-match warm-up and after the first half. Tests included rate of perceived exertion (RPE), 30-m sprint and countermovement (CMJ). (3) Results: Statistically significant differences were found (*p* < 0.001) when the measurements prior to the game were compared with those recorded after half time across all variables. Effect sizes (ES) were very large for RPE (ES = 1.82), moderate for 30-m sprint times (ES = 0.64) and small for CMJ (ES = −0.25). (4) Conclusions: After 45 min of official competition, our results suggest that U-16 soccer players demonstrated a reduction in sprint and jump performance, in addition to a higher RPE. Hence, this information could be useful when designing re-warming strategies that can be performed before the second half.

## 1. Introduction

Soccer is a team sport that requires high-intensity intermittent actions repeatedly [1], performed during two 45-min periods separated by a 15-min interval [2]. During a soccer match, it has been reported that players cover a total distance of 10 to 12 km [3,4]. Competition in soccer demands periods of activity that vary in intensity and duration, interspersed with periods of recovery where the player’s activity is low in intensity or static [5]. Thus, the ability to perform numerous high-intensity actions such as sprints, jumps and changes of direction, is generally considered an important factor for performance in soccer players [6,7]. These actions are the most frequent in the crucial moments of the matches, such as gaining possession of the ball, scoring or receiving a goal [8].

Fatigue can be defined as a decrease in performance caused by a reduction in the ability to apply force and accompanied by an increase in perceived exertion [9]. In previous research, Mohr et al. [1] reported a decline in performance during competitions which seems to occur in three different stages: after intense periods of brief duration, towards the end of the game and in the initial phase of the second half. The decrease in physical performance in the first minutes of the second half has been demonstrated both in the total distance covered [8,10,11] and in the distance covered at high and low intensity [4,10,11,12] compared to the same period corresponding to the first half. Due to this, various types of re-warm up strategies have been performed during half time [2,6,13,14,15,16,17,18,19], and are thereby applied as a method to attenuate the loss of performance in the initial minutes of the second half. Conversely, Mohr et al. [13] reported that the players who re-warmed up in half time had a greater performance decrement at the end of the game than the control players who did not. In this line, similar findings were reported by Zois et al. [16] in terms of a greater perceived fatigue after an intermittent protocol for players who performed a re-warm up. Therefore, due to this aforementioned controversy, it seems relevant to investigate the changes in physical performance between pre-game warm-up and before the second half in youth soccer players. In fact, this information will help coaches and physical trainers working in youth set-ups to make better decisions when deciding to perform a re-warm up protocol during half time (HT).

In terms of high-intensity runs, professional soccer players perform more runs during all stages of a match compared to non-professional soccer players, in addition to a greater sprinting and jumping ability [6,7]. Apart from high-intensity running, the ability of soccer players to produce explosive actions such as peak sprint and jump performance is also an important performance marker [7]. Hence, the assessment of jumping ability is useful because they reflect the capacity of the stretch-shortening cycle of the lower limbs musculature and the ability to indirectly assess muscle fatigue [20]. Furthermore, jump assessments are easy to administer without causing fatigue [21]. As such, the loss of jump height showed almost perfect relationships with blood lactate concentrations (r = 0.93–0.99) and ammonia (r = 0.94–0.99) when sprint training sessions were performed [22,23]. The large correlations observed between CMJ height loss and metabolic responses suggests that CMJ height could be used as a simple method to determine neuromuscular fatigue induced during sprint training [24].

With regards to rate of perceived exertion (RPE), this is a subjective measure, which has been shown to be a useful, valid and accessible tool to control training load [23,25]. RPE appears to be a good indicator of global internal load in soccer [25]. This method does not require expensive equipment, and it is very useful and practical for coaches to check the state of the players [25,26]. The Borg scale (0–10) is considered an indicator of exercise intensity that includes physiological (oxygen consumption, heart rate, ventilation, circulating glucose concentration and glycogen depletion) and psychological factors [27]. Although RPE has been shown to accurately reflect exercise intensity, it is possible that players may perceive the same physiological stimulus differently as a consequence of their individual psychological state [27,28]. Consequently, the use of RPE to monitor exercise intensity could be considered a valuable tool for detecting fatigue related to overtraining and reliably monitoring responses to training [26].

As mentioned above, to the best to our knowledge there is no scientific evidence that has assessed the physical performance changes to better determine youth soccer players’ physical fitness at the end of the first half during official competitions. Therefore, the aim of the study was to analyze whether 45-min of official competition may provoke a reduction in physical performance in youth soccer players. Considering the previous literature, we hypothesized that after 45-min of competition, changes in physical performance measures will be reported, and this is in line with previous studies that have indicated a performance decrement in linear sprint and vertical jump (CMJ) after 45 min during simulated or friendly soccer matches [6,29,30].

## 2. Materials and Methods

### 2.1. Participants

Twenty (*n* = 20) male youth soccer players from the Under-16 category (U-16) were recruited to participate in the present study (age: 14.4 ± 0.5 years, body mass: 65.1 ± 11.6 kg and height: 170 ± 0.05 cm). The team to which they belong participates in the fourth provincial category of the Andalusian Football Federation. All participants normally participate in three soccer training sessions per week lasting approximately 90 min, and an official competition during the weekends. Only the 10 players that the coach selected as starters in each game were assessed, while the goalkeepers and substitute players were excluded from the study.

### 2.2. Ethical Considerations

All participants were informed in detail about the content of the study, its objectives and possible risks and benefits. The tasks and tests performed in this study consisted of exercises that are usually done in training (sprints and jumps). Each player had a federative license, by which the parents at the beginning of the season signed an authorization document to participate in the club’s football activities. This type of intervention does not alter the normal football training or imply motor actions different from those of the usual practice of training sessions and matches [28,31]. Moreover, all participants were subjected to a medical examination prior to the beginning of the season and carried out the tests with no injuries or physical discomfort. Therefore, this study meets the requirements of the Declaration of Helsinki (WMA, 2013).

### 2.3. Design and Procedure

**Figure 1:**
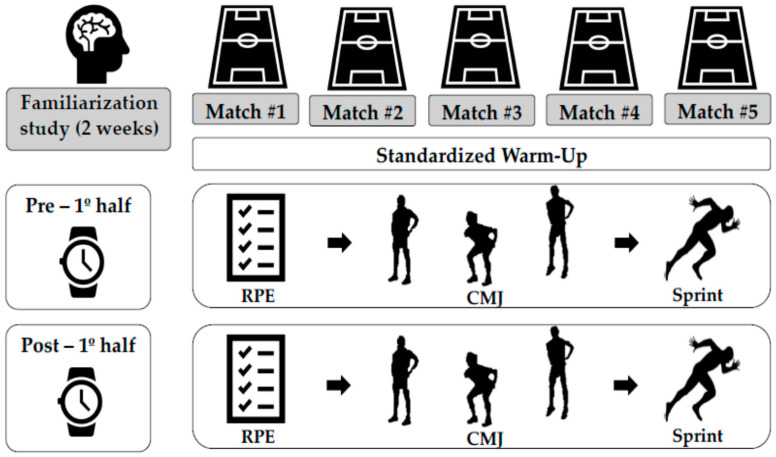
Study design.

A descriptive and cross-sectional study design was used. Data were collected from 5 official matches, of which 3 were home games and 2 were away. All the players had a process of test familiarization for 2 weeks prior to the first assessment, including the protocol and duration, which was done with the aim to avoid any delay that could affect the normal routine of players and coaches during half time. The week prior to the first match, a trial of all tests was carried out during a training session. From match 1 to 5, players performed the tests at two different moments: first, after the pre-first-half warm-up (Pre 1stH), and second, after the first half (Post 1stH, immediately after the referee signals at the end of the first half and before coaches talk). Each of the assessment moments included rate of perceived exertion (RPE), two 30-m sprints and two repetitions of CMJ. The tests were always performed in the same order, while all official matches were played at the Federations scheduled times (Figure 1). The pre-game warm-up was always done by a certified specialist strength and conditioning coach following the same protocol.

#### 2.3.1. Rate of Perceived Exertion

The RPE was registered 2-min after finishing Pre 1stH warm-up and 2-min after Post 1stH, and always before starting the physical tests. The RPE (Borg scale) was used with a scale of 0–10 following the steps previously reported [32]. The RPE values were provided verbally by each player and recorded on a spreadsheet designed for recording the information.

#### 2.3.2. Sprint Test

The 30-m sprint speed test was performed immediately after RPE, in dry weather conditions in an outdoor soccer pitch (4G artificial turf field). Photocell timing gates (Witty, Microgate, Bolzano, Italy) were placed at the start line (0-m), and at 30-m, so that the times to cover 0–30 m (T30) could be determined. A standing start with the lead-off foot placed 0.5-m behind the first timing gate was used. The best of both attempts was kept for further analysis. The players were also instructed not to make any type of backward movement with their upper body or feet, so as not to inadvertently trigger the first timing gate. This distance was selected, because it has been reported as the optimal distance to assess maximum sprint speed [33].

#### 2.3.3. Countermovement Jump Test

Because CMJ could be a very useful and consistent indirect measure of fatigue without the need to measure blood lactate or ammonia concentrations [23], this test was performed immediately after the sprint. The players performed two CMJ repetitions 90 s apart in the exact same order in which they had performed the sprints. In addition, the jumps were always carried out under the same conditions, inside the changing room over a platform made of rigid rubber. Jump height was obtained from flight time using an infrared platform (Optojump, Microgate, Bolzano, Italy). Before jumping, players were instructed to rest their hands on the hips while performing a downward movement followed by a maximum vertical jump. All players were instructed to keep their knees extended during the flight phase of the jump, and to land in an upright position to avoid the possibility of overestimating jump height, such that they maintain dorsiflexion of the ankles until landing [6,14,30,31].

### 2.4. Statistical Analysis

Data analysis and treatment was performed using SPSS software version 20.0 (SPSS Inc., Chicago, IL, USA) and presented as mean ± SD. The Shapiro–Wilk test was used to check the normal distribution of the data. To obtain the reliability of the measurements, Intraclass Correlation Coefficient (ICC) with a 95% confidence interval (95% CI) and coefficient of variation (CV) were used. To analyze the differences across all measured variables between Pre 1stH and Post 1stH, a Student’s *t*-test was used, while a Wilcoxon test was used in the case of RPE (non-parametric variable). Moreover, the magnitudes of the observed differences between the measurements were further analyzed by determining Cohen’s ‘d’ effect size (ES) with a 95% CI [34]. The following threshold values were used: trivial (0.0–0.19); small (0.2–0.59); moderate (0.6–1.1); large (1.2–1.9); and very large (>2.0) [35,36].

## 3. Results

**Figure 2:**
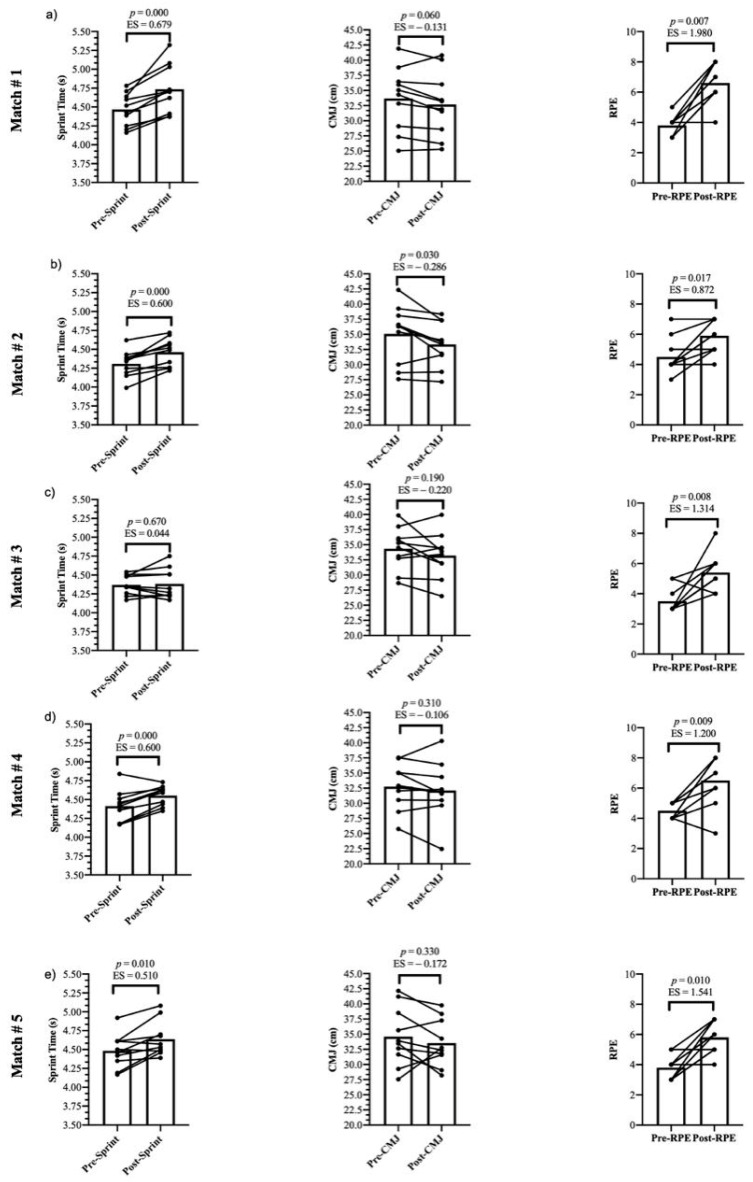
Match 1 to 5 (Figure **a**–**e**) physical and subjective responses from Pre 1stH to Post 1stH.

Data for sprints and CMJ were normally distributed (*p* > 0.05). Table 1 presents the descriptive data of the results obtained from Pre 1stH and Post 1stH, and the absolute and relative reliability of both sprints and CMJ tests of the five games. Comparisons between Pre 1stH and Post 1stH measures are presented in Table 2. The individual response in each of the five official matches of the soccer players is depicted in Figure 2, likewise match results are presented in Table 3. 

## 4. Discussion

To the best of our knowledge, this is the first study analyzing the physical responses in youth soccer players after 45-min of official competitions. Most of the research has been conducted in senior soccer players of various levels of competitions (from international elite to semi-professional). However, no studies to date have analyzed the acute effects in this specific age category. Thus, the aim of the present study was to determine if 45-min of official soccer competition may provoke a reduction in physical performance in U16 soccer players. Present findings indicate that there are significant differences (*p* < 0.001, ES small to large) across all variables from Pre 1stH to Post 1stH. Thus, at least in part, the results presented herein confirm our main hypothesis. Of note, caution must be taken as the individual match analysis showed that the responses to each particular game (and players) may vary due the multiplicity of factors that affect physical performance.

According to Edholm et al. [4], a 3% reduction in 10-m sprint performance (*p* < 0.05) was observed from Pre 1stH to Post 1stH in senior elite soccer players. Likewise, Robineau et al. [29] reported a significant reduction in sprint performance during the maximum velocity phase (i.e., 20–30 m split (3.2 ± 2.8%, *p* < 0.05)), accompanied by a significant reduction in maximal voluntary contraction of the quadriceps in both isometric and concentric contractions. These findings are in line with our study where the observed difference was also 3.2% (*p* < 0.001) (Table 2). Hence, a performance decrement at the end of the first half could be explained by the repetition of explosive-type efforts and soccer-specific actions, such as shooting or passing, accelerations, decelerations, etc. In contrast to this, in fourth division soccer players, no differences were indicated after the first half compared to Pre 1stH in 30-m sprint tests [1,37]. In this sense, it has been mentioned that the existing differences in physical performance of sub-elite compared to elite soccer players (senior) could be the main reason behind the reductions observed in sprint performance [6]. On the other hand, Meckel et al. [38] indicated that after performing 12 repeated sprints over 20-m, no differences were found in the results obtained pre to post first-half. Overall, the controversies can be explained by the fact that most of the aforementioned studies were conducted in senior elite, professional or semi-professional players. As such, position, age category and playing level have been reported to represent different physiological backgrounds [39].

With regards to jumping ability, Oliver et al. [30] observed a reduced CMJ height (*p* < 0.05) after a simulated first-half. These results coincide with the results of the present study, where a reduced CMJ height was observed from Pre 1stH to Post 1stH (3.3%, *p* < 0.001) (Table 2). Reductions in CMJ height could be considered as a good indicator of fatigue in the absence of direct laboratory-based measurements, as it could be useful to obtain information about acute responses through an actual field-based measurement in order to obtain practical information related to neuromuscular fatigue provoked [22]. Beyond that, it is difficult to compare our results with those provided by Oliver et al. [30] due to the protocol used, which was based on a simulated soccer game’s demands on a treadmill. It is important to highlight and consider that, to the authors knowledge, it is not possible to compare a simulated soccer game with the reality of an official match, regardless of whether the mean age of the participants was similar to that of our study. In fact, the players’ movement patterns and exercise-induced fatigue could largely differ from real-game situations. Moreover, Edholm et al. [4] indicated that after assessing CMJs (followed by two 10-m sprints), there were no differences between the values obtained from Pre 1stH to Post 1stH. These results do not coincide with those reported in our study, since our results indicate that there are differences in the CMJ between the moments before and after the first half. Neither Fashioni et al. [14] nor Robineau et al. [29] obtained statistically significant differences after comparing the CMJ from Pre 1stH to Post 1stH. Thus, the youth players in our study may have had a greater physical demand during the first half, and in turn, this may have caused a reduction in CMJ performance. Nonetheless, further studies are needed to clarify these aspects.

Regarding changes in the perceived effort, Meckel et al. [38] indicated that there are significant differences in RPE from Pre 1stH to Post 1stH (*p* < 0.01) when a repeated sprint protocol is performed either before or after. These data are in line with the results of our study since we also obtained significant differences when we compared the RPE from Pre 1stH to Post 1stH. (*p* < 0.001). Conversely, Fashioni et al. [14] reported that no differences were found in RPE when comparing the pre and post first half RPE measures. Like heart rate and blood lactate concentration in capillary blood [40,41], the control of RPE has been shown as a valid tool to quantify the internal training load, since a double dimension, physical and psychological, is present in athletes of different performance levels [25]. It is therefore important to consider the higher demands of soccer when compared to other team sports, as it was reported that soccer has the largest total running distances [42]. This finding confirms the higher running demands of a competitive soccer game with a concomitant increase in RPE measures after 45 min of official competition. In fact, RPE was the only variable that showed a similar pattern across all five games (ES = −0.87 to −1.54; *p* < 0.01) (please see Figure 2).

In the global analysis of the five games (Table 2), it was observed that after the first 45-min of official competition, there was a decrease in sprint performance and jumping ability with an increase in RPE. However, in the analysis of each match and the individual response of the soccer players (Figure 2), a different trend was observed. Even in some matches, the pre–post difference in CMJ and the 30-m sprint was not significant (*p* ≥ 0.05, ES trivial). These diverse changes observed between soccer players and matches could be justified by the fact that soccer matches can vary depending on environmental (i.e., temperature, altitude, etc.), tactical (i.e., ball possession, etc.) and situational (i.e., home or away games, partial score of the match, etc.) factors, and as a consequence, the physical performance of the players may be affected differently [43]. Furthermore, some of these factors seem to affect players in distinct ways depending on the specific position, since for example, central and lateral defenders increased the total distance covered and the distances covered in running at medium, high and very high speed and sprints when their team was losing, while the attackers showed the opposite trend [44]. Overall, it is important to consider a more comprehensive test battery that could better reflect and identify these changes in physical performance, while in turn this may help to decide which strategy (re-warm up or not) must be used prior to the commencement of the second half.

The main difference between the present study and the rest of the studies that have assessed performance using the same or similar tests, is that the present study has been carried out in official matches, whereas the others have focused on friendly or simulated matches. This description is of great importance, since we found this difference to be one of the main reasons behind the reported differences in results between studies.

Additionally, the present study is limited by its cross-sectional design and the selected performance measures, since it is important to consider a larger number of direct (e.g., blood lactate, ammonia, creatin kinase) and indirect (e.g., strength, acceleration, various jump tasks) assessments to better understand how soccer players respond after 45 min during official competitions. Therefore, due to the limited time available (15 min), we understand that there was no additional time to include such measures. Future studies using similar methods should include a larger sample size (larger number of matches and players). Lastly, the authors acknowledge that the present study does not add much information to the existing knowledge about fatigue and recovery in soccer, although it is important to consider the practical applications derived from the results obtained during real-life scenarios (i.e., competitions).

## 5. Conclusions

The results obtained show that after 45-min of official competition in U-16 soccer players, there is a decrease in sprint performance and jumping ability, along with an increase in RPE. In this sense, youth soccer players perceived a greater effort at the end of the first half of each game. Finally, caution must be taken as the individual analysis showed that the response to each particular match may be different.

## 6. Practical Applications

These results provide relevant information regarding the physical performance in which young soccer players finish the first 45 min during a competitive match, and therefore could be useful when designing re-warming strategies that can be performed before the second half. Additionally, coaches may consider the use of CMJ, sprint and RPE as simple and quick options to quantify changes in physical fitness after 45-min of competition in young soccer players, while this information may be used to decide the implementation of re-warm up strategies to improve soccer players’ performance.

## Figures and Tables

**Table 1:** Physical performance and reliability data obtained during the official matches.

Variables	Pre 1st Half				Post 1st Half			
	Mean ± SD	CV%	CI (95%)	ICC	Mean ± SD	CV%	CI (95%)	ICC
RPE (0–10)	4.02 ± 0.89	-	(3.7–4.2)	-	6.04 ± 1.27	-	(5.6–6.4)	-
Sprint (s)	4.40 ± 0.2	1.5	(4.3–4.5)	0.94	4.55 ± 0.25	1.5	(4.5–4.6)	0.94
CMJ (cm)	34.10 ± 4.57	5.8	(33.1-34.9)	0.89	32.98 ± 4.30	5.5	(32.1–33.8)	0.90

Note: CV%: coefficient of variation; ICC: intraclass correlation coefficient; CI 95%: 95% confidence interval.

**Table 2:** Between-moments comparison and changes in the physical performance during official soccer matches.

Variables	∆ ± SD	%Δ	*p* Value	ES	Magnitude
RPE	2.02 ± 1.37	33.4	<0.001	1.85	Large
Sprint 30 m (s)	0.15 ± 0.14	3.2	<0.001	0.64	Moderate
CMJ (cm)	−1.12 ± 2.25	−3.3	<0.001	−0.25	Small

Note: Data are presented as mean differences (M ± SD); %∆: percentage of change; ES: Cohen’s ‘d’ effect size; Magnitude: ES based on the Hopkins criteria.

**Table 3:** Descriptive characteristics derived from each official match.

Temperature	Match Number	Home/Away	Score at 1stH	Final Score
21 °C	1st	Away	0–4	1–11
20 °C	2nd	Local	4–0	11–4
25 °C	3rd	Local	4–0	9–2
21 °C	4th	Local	3–0	6–2
23 °C	5th	Away	0–1	1–3

## Data Availability

The data presented in this study are available upon request to the corresponding author. The data are not publicly available due to privacy.
